# Urinary volatile organic compounds and stroke risk: A cross-sectional analysis of NHANES data

**DOI:** 10.1097/MD.0000000000048786

**Published:** 2026-05-15

**Authors:** Hanguang Liu, Xiaoying Zhu

**Affiliations:** a Department of Neurology, Beilun People’s Hospital, Ningbo, Zhejiang, China.

**Keywords:** Bayesian kernel machine regression (BKMR), diabetes and stroke, stroke risk, volatile organic compounds (VOCs), weighted quantile sum regression (WQS)

## Abstract

Volatile organic compounds (VOCs) are ubiquitous environmental pollutants. However, the effect of ambient VOCs, alone or in combination, on stroke incidence in the general population remains unclear and warrants further investigation. Urinary VOC metabolites were assessed using data from the National Health and Nutrition Examination Survey for the years 2011 and 2020. Three statistical techniques were used: weighted logistic regression, weighted quantile sum regression, and Bayesian kernel machine regression (BKMR). Mediation analysis was also carried out to identify potential mediating effects. In univariate weighted logistic regression, the metabolites CEMA, 3HPMA, and DHBMA showed significant associations with stroke. However, both weighted quantile sum and BKMR analyses did not identify a significant association between VOC mixtures and stroke risk in the overall population. In subgroup analyses, BKMR suggested a positive association between VOC mixtures and stroke risk only among individuals with diabetes, with DHBMA being the main contributor. No significant interactions between individual VOCs were observed in the total population. Several VOC metabolites, including *N*-acetyl-S-(2-carboxyethyl)-l-cysteine (CEMA), 3-hydroxypropyl mercapturic acid (3HPMA), and *N*-acetyl-S-(3,4-dihydroxybutyl)-l-cysteine (DHBMA), were associated with stroke risk, but no overall mixture effect was observed in the general population. The association was limited to the diabetic subgroup and warrants cautious interpretation and further longitudinal validation.

## 1. Introduction

Stroke is a major global health problem and the second leading cause of disability and death worldwide. There are 2 main types of strokes: ischemic and hemorrhagic. In 2020, there will be an estimated 12.2 million new cases of stroke worldwide, resulting in approximately 6.55 million deaths. Of these deaths, approximately 3.5 million will be attributed to ischemic stroke and 3.05 million to hemorrhagic stroke. In the United States, it is estimated that approximately 8,00,000 people suffer a stroke each year, of which approximately 6,10,000 are first-time sufferers. Air pollution contributes to approximately 16.9% of the global stroke burden, with increasing evidence pointing to its role as a novel risk factor for stroke.^[1]^ Air pollution, characterized by particulate matter, sulphates, ozone, nitrous oxide and volatile organic compounds (VOCs), can exacerbate conditions conducive to stroke.

VOCs are a major class of gaseous compounds found in the air of many residential and commercial environments.^[2]^ Human exposure to VOCs has been increasing worldwide.^[3]^ VOCs are emitted from both natural and anthropogenic sources. Natural sources include emissions from vegetation, forest fires and anaerobic marsh processes. Anthropogenic activities also release VOCs from domestic and industrial activities such as food production, fertilizer and pesticide use, and septic tank systems. Even low levels of exposure to VOCs can cause adverse health effects.^[4]^ Studies have shown that exposure to VOCs is associated with neurological, developmental, systemic, immune, reproductive and genotoxic effects in animal models and humans. A study conducted in China found that VOCs were an independent risk factor for all-cause mortality in stroke patients.^[5]^ Another study conducted in the United States suggested that VOCs are associated with an increased risk of nonfatal cardiovascular and cerebrovascular events, including stroke, in American adults.^[6]^ Real-world studies often involve simultaneous exposure to multiple air pollutants, so it is important to estimate the individual effects of each pollutant as well as their combined effects when studying VOCs.

VOCs may indirectly or directly induce cardiovascular damage through various mechanisms, including induction of insulin resistance, alteration of blood pressure regulation, effects on lipoprotein function, acceleration of circulating endothelial progenitor cell depletion, systemic dyslipidemia and damage to the cardiovascular endothelium.^[7]^ Dyslipidemia is a recognized risk factor for ischemic stroke, with the coexistence of high triglyceride (TG) levels and either high low-density lipoprotein cholesterol (LDL-C) or low high-density lipoprotein cholesterol (HDL-C) further increasing the risk of stroke.^[8,9]^ Mediation effect analysis is a method used to explore an etiological mechanism. Therefore, this study used mediation analysis to investigate whether VOCs contribute to the risk of stroke occurrence by influencing dyslipidemia.

While the health effects of VOC exposure are becoming more widely recognized, there remains a gap in the literature regarding its association with stroke, particularly in large, population-based studies. To address this gap, we conducted a cross-sectional analysis using data from the National Health and Nutrition Examination Survey (NHANES). This study aimed to investigate the relationship between VOC exposure and stroke risk, controlling for individual and environmental confounding factors. By utilizing the large and diverse NHANES dataset, we seek to contribute to a better understanding of how environmental exposures like VOCs may influence stroke risk, potentially informing public health policies aimed at reducing stroke incidence.

## 2. Materials and methods

### 2.1. Research population

This study utilized data from the NHANES from 2011 to 2020, comprising 45,462 participants. Of these, 11,147 had VOC measurements. After excluding 6208 participants with missing covariates, 42 pregnant women, and 5 participants with incomplete stroke data, the final analysis included 4892 participants. The detailed participant selection process is illustrated in Figure S1. All participants provided informed consent, and the NHANES protocol was approved by the National Center for Health Statistics Research Ethics Review Board. As the study used publicly available de-identified data, no additional ethical approval was required.

### 2.2. Definition of stroke

Data were collected via a questionnaire that asked whether candidates aged 20 and over had ever been diagnosed with a stroke by doctors or other healthcare professionals. A positive response to this question served as confirmation of a stroke diagnosis.

### 2.3. Pollutant measurement

VOC exposure was assessed using urinary VOC metabolites as surrogates due to their longer detection time compared to blood. For concentrations below the limit of detection (LOD), values were replaced by the square root of LOD divided by 2. VOCs with over one-third of measurements below the LOD were excluded. Data on 15 VOCs from NHANES were used to examine their association with stroke risk, including *N*-acetyl-S-(2-carboxyethyl)-l-cysteine (CEMA), *N*-acetyl-S-(3,4-dihydroxybutyl)-l-cysteine (DHBMA), and *N*-acetyl-S-(3-hydroxy-1-methylpropyl)-l-cysteine (MHBMA3).

### 2.4. Covariate

Covariates considered in this study,^[8]^ based on previous research, include age, sex, race/ethnicity, education level, poverty-income ratio, body mass index (BMI), serum cotinine levels, alcohol consumption, physical activity, energy intake, hypertension, and diabetes. Age and BMI were treated as continuous variables, while categorical variables were classified as follows: sex (male, female), ethnicity (Mexican American, other Hispanic, non-Hispanic white, non-Hispanic black, etc), education level (<9th grade, 9th–11th grade, high school, some college or associate degree, college and above), poverty-income ratio (≤1.30, 1.31–3.50, >3.50), alcohol consumption (≥12 drinks/yr), physical activity (low, medium, high), and energy intake (low, adequate, high). To accurately determine smoking status and assess exposure to environmental tobacco smoke, serum cotinine concentrations were used instead of relying solely on self-reported questionnaire data on smoking habits.

### 2.5. Statistical methods

Descriptive statistics summarized participant demographics and biomarker levels. Continuous variables were expressed as medians with interquartile ranges (IQRs) and compared using the Mann–Whitney *U* test, while categorical variables were presented as frequencies and compared using the chi-squared test.

Prior to statistical analysis, urinary mVOC concentrations were normalized to urinary creatinine and subjected to natural logarithmic transformation (ln transformation) to obtain a normal distribution. Relationships between chemicals were assessed using Spearman correlation. The chemicals were quartile-transformed, and a univariate weighted logistic regression was conducted to examine the association between creatinine-standardized VOCs and stroke, with subgroup analyses based on hypertension and diabetes status.

Weighted quantile sum regression (WQS) and Bayesian kernel machine regression (BKMR) were used to analyze the association between VOC mixtures and stroke. WQS assigned weights to each VOC to determine their relative importance in the mixture and derived confidence intervals (CI) and P values using 5000 bootstrap iterations. BKMR quantified the overall exposure–response relationship and the contribution of individual VOCs through 50,000 iterations of a Markov Chain Monte Carlo sampler, generating posterior inclusion probabilities (PIPs).

Mediation analysis assessed the role of TG and HDL-C as mediators in the VOC-stroke relationship. Direct effects reflected VOC exposure’s independent influence on stroke, while indirect effects captured the influence mediated by TG and HDL-C. The mediator ratio was calculated by dividing the indirect effect by the total effect.

All analyses were conducted in R (version 4.3.1; The R Foundation for Statistical Computing, https://cran.r-project.org/) using the survey, gWQS, bkmr, mediation, and BruceR packages. Statistical significance was set at *P* < .05. Statistical significance was set at *P* < .05. To rigorously assess the stability of our findings, multiple robustness checks and sensitivity analyses were performed. First, we modeled VOC metabolites both as categorical variables (quartiles) and continuous variables (ln-transformed). Second, the deliberate application of 2 distinct modeling frameworks – WQS and BKMR – served as an internal methodological robustness check to evaluate the consistency of mixture effects across different statistical assumptions.

## 3. Results

### 3.1. Baseline features

The characteristics of the study population are shown in Table 1. A total of 4892 participants were included in the analysis, with a stroke incidence rate of 3.78%. There were significant differences in age, race, education level, marital status, family income, BMI, energy intake, history of hypertension and diabetes between the stroke and non-stroke groups.

**Table 1:** Characteristics of study population (N = 4892), NHANES, United States, 2011 to 2020.

	Stroke	Non-stroke	*P*
	N = 4707	N = 185	
Age, yr	48.0 [33.0;62.0]	66.0 [57.0;74.0]	**<.001**
Gender, n (%)			.243
Male	2427 (51.6%)	104 (56.2%)	
Female	2280 (48.4%)	81 (43.8%)	
Race, n (%)			**<.001**
Mexican American	570 (12.1%)	11 (5.95%)	
Other Hispanic	495 (10.5%)	13 (7.03%)	
Non_Hispanic White	1885 (40.0%)	79 (42.7%)	
Non_Hispanic Black	1085 (23.1%)	64 (34.6%)	
Other race	672 (14.3%)	18 (9.73%)	
Education, n (%)			**<.001**
Less than 9th grade	318 (6.76%)	22 (11.9%)	
9_11th grade	523 (11.1%)	31 (16.8%)	
High school grade	1076 (22.9%)	58 (31.4%)	
Some college or AA degree	1517 (32.2%)	53 (28.6%)	
College graduate or above	1273 (27.0%)	21 (11.4%)	
Marital, n (%)			**.001**
Married	2482 (52.7%)	94 (50.8%)	
Widowed	557 (11.8%)	46 (24.9%)	
Divorced	610 (13.0%)	29 (15.7%)	
Separated	86 (1.83%)	2 (1.08%)	
Never married	679 (14.4%)	10 (5.41%)	
Living with partner	293 (6.22%)	4 (2.16%)	
Family PIR, n (%)			**<.001**
≤1.30	1409 (29.9%)	76 (41.1%)	
1.31–3.5	1756 (37.3%)	74 (40.0%)	
>3.5	1542 (32.8%)	35 (18.9%)	
Cotinine category, n (%)			.205
	1487 (31.6%)	49 (26.5%)	
	2027 (43.1%)	80 (43.2%)	
	1193 (25.3%)	56 (30.3%)	
Alcohol, n (%)			.075
Yes	1548 (32.9%)	73 (39.5%)	
No	3159 (67.1%)	112 (60.5%)	
BMI, kg/m^2^	28.2 [24.5; 33.1]	29.6 [26.0; 33.9]	**.013**
Activity, n (%)			.879
No or lower	2623 (55.7%)	105 (56.8%)	
Moderate	1041 (22.1%)	38 (20.5%)	
Vigorous	1043 (22.2%)	42 (22.7%)	
Energy intake, n (%)			**<.001**
Low	1863 (39.6%)	104 (56.2%)	
Adequate	1954 (41.5%)	60 (32.4%)	
High	890 (18.9%)	21 (11.4%)	
Hypertension, n (%)			**<.001**
Yes	3083 (65.5%)	47 (25.4%)	
No	1624 (34.5%)	138 (74.6%)	
Diabetes, n (%)			**<.001**
Yes	4077 (86.6%)	122 (65.9%)	
No	630 (13.4%)	63 (34.1%)	

Bold values indicate statistically significant results at the .05 significance level (*P* < .05).

BMI = body mass index, CI = confidence interval, HDL-C = high-density lipoprotein cholesterol, OR = odds ratio, PIR = poverty-income ratio, TG = triglycerides, VOC = volatile organic compounds.

### 3.2. Chemical exposure measurement and its correlation

**Figure 1.**
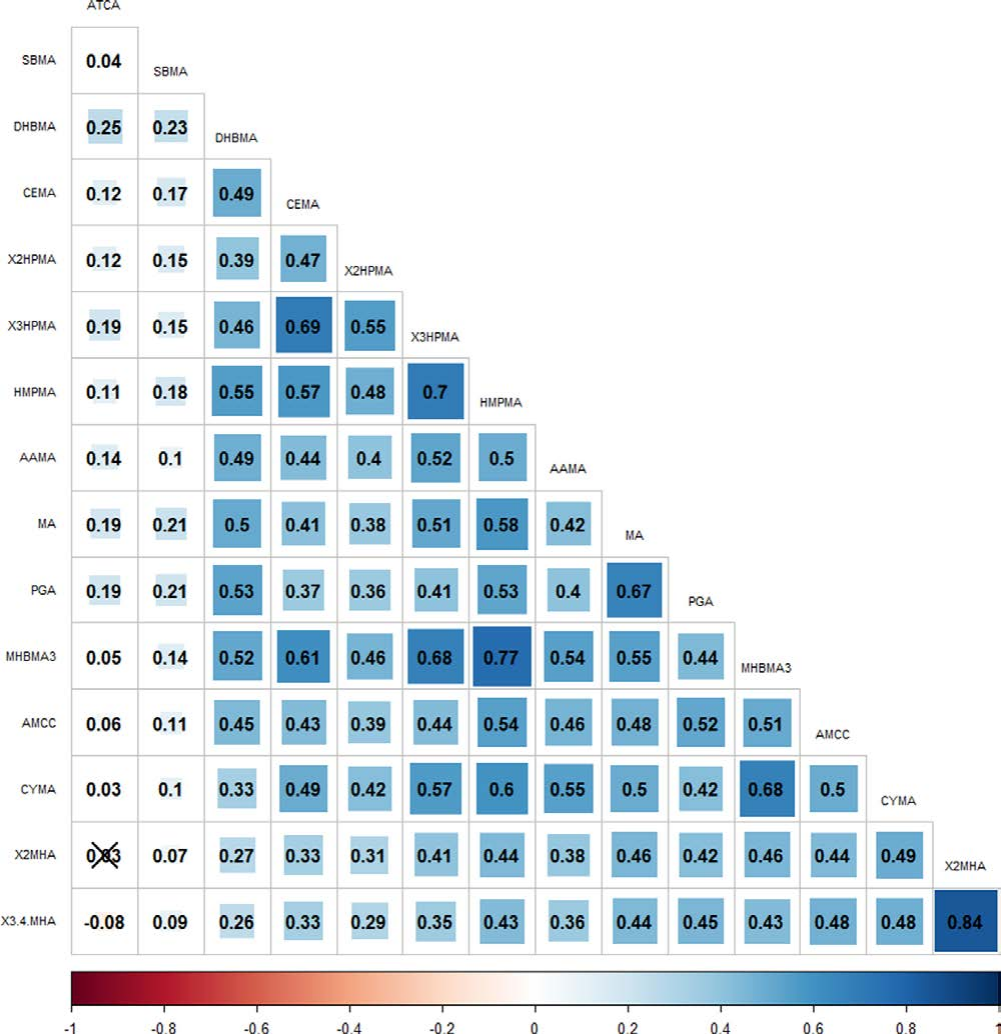
Spearman correlation coefficient between pairwise chemical measurement values.

Tables S1 and S2, show the detection rate and distribution of VOCs among the participants. Fifteen chemicals had a detection rate of more than two thirds in all participants. Spearman correlation coefficients were calculated pairwise for these 15 chemicals. Figure 1 shows that the highest correlation coefficient (0.84) was between 2MHA and 3.4MHA, indicating a strong positive correlation. Other significant correlations were observed between MNMBA3 and HMPMA (0.77) and X3HPMA and HMPMA (0.70). Several other pairs of VOCs also showed moderate to strong correlations, suggesting that these chemical exposures are related.

### 3.3. Univariate weighted logistic regression

Urinary creatinine-corrected VOC concentration was analyzed as a continuous variable by weighted logistic regression. VOC concentrations were divided into quartiles according to their natural log values, and the lowest quartile was used as the control group in the regression analysis. As shown in Table 2, after adjustment for covariates, log-transformed concentrations of CEMA and 3HPMA were significantly associated with stroke risk (OR = 1.29, 95% CI = 1.04–1.60; OR = 1.22, 95% CI = 1.01–1.48). In addition, DHBMA concentration in the third quartile was significantly associated with stroke compared to that in the lowest quartile (OR = 2.08, 95% CI = 1.09–4.00). Tables S3 and S4, show the results of the weighted logistic regression analysis of VOCs in the hypertensive and diabetic populations, respectively.

**Table 2:** Association of individual VOCs with stroke in the total population.

	Ln-transform		Quartile1	Quartile2		Quartile3		Quartile4	
	OR (95% CI)	*P*		*OR (95%CI*)	*P*	*OR (95%CI*)	*P*	*OR (95%CI*)	*P*
CEMA	1.29 (1.04–1.60)	**.022**	Reference	0.99 (0.51–1.92)	0.965	1.68 (0.91–3.11)	.096	1.75 (0.95–3.23)	0.07
3HPMA	1.22 (1.01–1.48)	**.037**	Reference	1.44 (0.74–2.81)	0.271	2.06 (1.05–4.02)	**.035**	1.94 (1.02–3.68)	**0.043**
AAMA	1.19 (0.91–1.55)	.197	Reference	1.78 (0.97–3.27)	0.063	1.61 (0.83–3.15)	.158	1.31 (0.69–2.47)	0.397
CYMA	1.02 (0.89–1.18)	.734	Reference	1.30 (0.77–2.17)	0.318	1.37 (0.69–2.75)	.360	1.16 (0.57–2.34)	0.672
HMPMA	0.99 (0.76–1.28)	.914	Reference	1.22 (0.56–2.63)	0.612	1.06 (0.53–2.13)	.859	1.27 (0.58–2.80)	0.543
ATCA	1.07 (0.93–1.24)	.355	Reference	0.95 (0.51–1.79)	0.871	1.94 (1.18–3.19)	**.010**	1.03 (0.54–1.95)	0.931
PGA	1.38 (0.98–1.96)	.067	Reference	1.63 (0.84–3.15)	0.141	1.41 (0.72–2.78)	.306	1.72 (0.80–3.72)	0.161
MA	0.97 (0.70–1.36)	.864	Reference	1.31 (0.71–2.41)	0.376	1.52 (0.77–3.02)	.219	0.96 (0.46–1.99)	0.909
AMCC	1.14 (0.83–1.56)	.397	Reference	0.79 (0.36–1.77)	0.566	1.54 (0.64–3.67)	.323	1.44 (0.56–3.74)	0.444
2HPMA	1.12 (0.82–1.52)	.481	Reference	1.01 (0.53–1.93)	0.965	0.80 (0.43–1.52)	.493	1.15 (0.52–2.51)	0.728
SBMA	0.93 (0.79–1.10)	.391	Reference	1.85 (1.01–3.41)	**0.048**	1.21 (0.69–2.11)	.500	0.92 (0.58–1.46)	0.730
2MHA	1.09 (0.86–1.37)	.481	Reference	1.06 (0.56–1.99)	0.859	1.49 (0.71–3.13)	.280	1.54 (0.70–3.41)	0.275
3,4.MHA	1.10 (0.87–1.39)	.426	Reference	1.69 (0.93–3.06)	0.082	1.64 (0.76–3.55)	.199	1.95 (0.86–4.40)	0.107
DHBMA	1.11 (0.72–1.70)	.636	Reference	1.87 (1.03–3.41)	**0.041**	2.08 (1.09–4.00)	**.028**	1.43 (0.79–2.61)	0.233
MHBMA3	1.03 (0.79–1.34)	.808	Reference	1.82 (0.92–3.62)	0.085	1.08 (0.55–2.13)	.812	1.84 (0.75–4.50)	0.175

Bold values indicate statistically significant results at the .05 significance level (*P* < .05).

2HPMA = 2-hydroxypropyl mercapturic acid, 2MHA = 2-methylhippuric acid, 3,4.MHA = 3,4-methylhippuric acid, 3HPMA = 3-hydroxypropylmercapturic acid, AAMA = *N*-Acetyl-S-(2-carbamoylethyl)-l-cysteine, AMCC = *N*-Acetyl-S-(2-carboxy-2-methylpropyl)-l-cysteine, ATCA = *N*-Acetyl-S-(2-amino-2-thioxoethyl)-l-cysteine, CEMA = *N*-Acetyl-S-(2-carboxyethyl)-l-cysteine, CYMA = *N*-Acetyl-S-(2-cyanoethyl)-l-cysteine, DHBMA = dihydroxybutyl mercapturic acid, HMPMA = S-(3-hydroxy-l-methylpropyl) mercapturic acid, MA = mandelic acid, MHBMA3 = monohydroxybutenyl mercapturic acid, PGA = phenylglyoxylic acid, SBMA = S-benzylmercapturic acid.

### 3.4. Weighted quantile sum (WQS) regression

**Figure 2.**
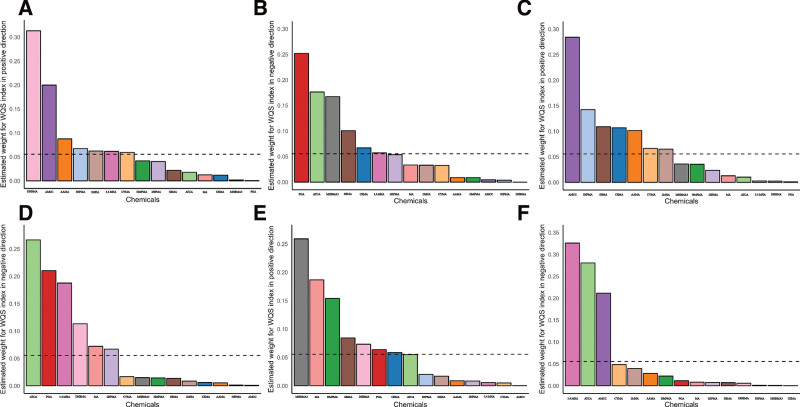
Weighted quantile sum (WQS) regression index weights for stroke risk across different populations; This figure presents the estimated weights of various volatile organic compounds (VOCs) contributing to stroke risk as calculated by the WQS regression model; (A, B) Weights for the total population under positive (A) and negative (B) direction constraints; (C, D) Weights for hypertensive populations under positive (C) and negative (D) direction constraints; (E, F) Weights for diabetic populations under positive (E) and negative (F) direction constraints. VOC = volatile organic compound, WQS = weighted quantile sum.

The association between urinary creatinine-corrected VOC concentrations and stroke was assessed using WQS regression. In the total population, no significant association was observed under either positive or negative direction constraints (Fig. 2A, B).

In the hypertensive population, no significant association was observed, with AMCC, 3HPMA, and SBMA showing relatively higher weights (Fig. 2C, D).

In the diabetic population, the mixture index was also not significant in either the positive or negative constraint models, but MHBMA3, MA, and HMPMA (positive constraint), as well as 3,4-MHA, ATCA, and AMCC (negative constraint), had higher relative weights (all > 1/18), indicating their relative importance in this subgroup (Fig. 2E, F, Table S5).

These results highlight the key VOC components contributing to stroke risk in each subgroup, without overloading the text with redundant details.

### 3.5. Bayesian kernel machine regression (BKMR)

**Figure 3.**
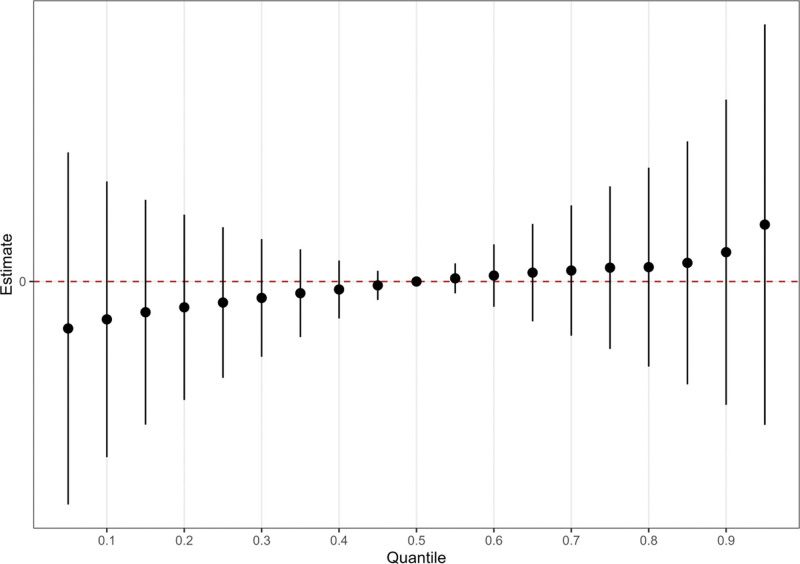
In the BKMR model, the 95% CI estimates the joint effect of VOC mixture exposure on stroke for the entire population, comparing VOC concentrations at specific percentiles (from 0.05 to 0.95, increasing by 0.05) to their 50th percentile. The model is adjusted for age, sex, race, education level, marital status, poverty-income ratio, body mass index, serum cotinine levels, alcohol consumption, physical activity, energy intake, hypertension status, and diabetes status. VOC = volatile organic compound.

The BKMR model was used to assess the association between urinary creatinine-corrected VOC concentrations and stroke risk. The results showed that higher levels of VOC mixtures were not significantly associated with an increased risk of stroke in the total population (Fig. 3).

**Figure 4.**
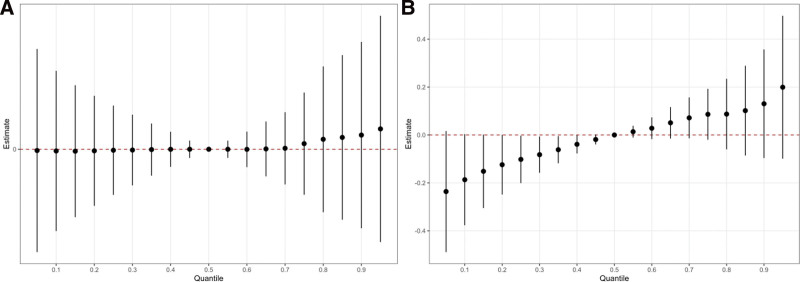
In the BKMR model, the 95% CI estimates the joint effect of VOC mixture exposure on stroke for individuals with hypertension (A) and diabetes (B), comparing VOC concentrations at specific percentiles (from 0.05 to 0.95, increasing by 0.05) to their 50th percentile. Model (A) is adjusted for age, sex, race, education level, marital status, poverty-income ratio, body mass index, serum cotinine levels, alcohol consumption, physical activity, energy intake, and diabetes status. Model (B) is adjusted for age, sex, race, education level, marital status, poverty-income ratio, body mass index, serum cotinine levels, alcohol consumption, physical activity, energy intake, and hypertension status. VOC = volatile organic compound.

No significant association was observed between VOC mixtures and stroke risk in the hypertensive population (Fig. 4A). However, a positive dose–response relationship was observed between VOC mixtures and stroke risk in the diabetic population (Fig. 4B). The ORs for stroke at the 25th and 75th percentiles were 0.90 (95% CI: 0.82–0.99) and 1.09 (95% CI: 0.98–1.21), respectively.

DHBMA had the highest posterior inclusion probability (PIP, a measure of the relative importance of each exposure in the BKMR model; PIP = 0.644), suggesting that it played an important role in mediating the association between VOC mixtures and stroke risk in the diabetes population (Table S6). For the whole population, we examined the exposure–response function of 1 chemical with stroke risk by fixing other chemicals at median levels and varying the exposure levels of the second chemical at different percentiles. The results showed no interaction between 2 chemicals on the total stroke risk of the population (Fig. S2).

### 3.6. Analysis of lipid mediation of VOCs and stroke

We tested whether TG and HDL-C mediate the association between VOC exposure and stroke. Mediation models were used to test the direct and indirect effects of VOC exposure on stroke risk, with TG and HDL-C as mediators. The pathways for TG-HDL-C and HDL-C-TG were tested both separately and sequentially. As shown in Figures S3 and S4 and Tables S7–S14, Supplemental Digital Content, neither TG nor HDL-C significantly mediated the association between VOC exposure and stroke. The direct effect of VOC exposure on stroke remained significant, whereas the indirect effects through TG and HDL-C were not statistically significant.

These results indicate that TG and HDL-C do not play a significant role in mediating the relationship between VOC exposure and stroke risk, suggesting that other mechanisms may be involved.

## 4. Discussion

In this study, CEMA, 3HPMA and DHBMA were identified as the most important contributors to stroke. No overall effect of VOCs on stroke was observed in the total population. However, there was a clear upward trend in the risk of stroke with increasing levels of the VOC mixture, particularly in the diabetic group, with DHBMA being the main factor driving this association.

3HPMA and CEMA are metabolites of acrolein, which is produced by tobacco smoke, industrial emissions and cooking. Acrolein exposure has been shown to cause cardiovascular toxicity by impairing endothelial function and increasing the risk of hypertension in individuals with increased sympathetic nervous activity.^[10]^ Acrolein exposure has been shown to increase blood pressure in normotensive and hypertensive rats.^[11,12]^ As hypertension is a known risk factor for stroke, it is possible that acrolein and its metabolites contribute to the development of stroke by aggravating hypertension. Acrolein exposure causes endothelial dysfunction by inhibiting activation of endothelial nitric oxide synthase, inhibiting endothelial cell migration, blocking vascular endothelial growth factor and reducing circulating angiogenic cells.^[10]^ In addition, oxidative stress caused by acrolein activates pro-inflammatory pathways, causing vascular inflammation, which may promote the development of atherosclerosis and increase the risk of stroke.

The metabolites DHBMA and MHBMA3 derived from 1,3-butadiene are often associated with industrial activities such as rubber production and combustion processes. Studies have shown that exposure to 1,3-butadiene can accelerate the formation of atherosclerotic lesions in animal models. In addition, polymorphisms in phase II enzymes involved in the metabolism of 1,3-butadiene have been associated with human atherosclerosis.^[13]^ A history of atherosclerosis significantly increases the risk of recurrent ischemic stroke. Risk factors for recurrent ischemic stroke include the presence of vulnerable, complex, ulcerated, unstable plaques and plaque size.

The presence of urinary metabolites of 1,3-butadiene and the associated cardiovascular risk may be partly due to a reduction in early progenitor cells. These cells play an important role in vascular repair and in preventing the progression of atherosclerosis.^[14]^ Exposure to total VOCs may have a cumulative adverse effect by stimulating angiogenesis in cells. Therefore, exposure to 1,3-butadiene and its metabolites may contribute to the development of ischemic stroke by accelerating atherosclerosis. In addition, exposure to 1,3-butadiene may increase the risk of hypertension in individuals with high sympathetic tone.^[10]^ This mechanism is one of the ways in which 1,3-butadiene promotes the development of stroke. Acrolein and 1,3-butadiene, together with their metabolites, have similar mechanisms for promoting stroke.

Exposure to a single VOC is rare and unrealistic because many VOCs are emitted from similar sources and have similar toxicological effects. It is therefore necessary to study and assess exposure to mixtures of VOCs. Although no synergistic effect of VOCs on stroke was found in this study, other studies have shown that the effects of VOCs on cardiovascular and cerebrovascular disease are mixed.^[15]^ The complex relationship between mixtures of VOCs and the cardiovascular and cerebrovascular systems and their different classifications are important for understanding the association.

In the BKMR analysis conducted on the diabetic cohort, an elevated concentration of the VOC mixture was found to be significantly correlated with an increased risk of stroke, with DHBMA being a major contributor to this association. The exact mechanism by which VOCs exacerbate stroke in diabetic patients remains elusive. However, previous research has shown that diabetes is an independent risk factor for recurrent stroke in people with ischemic stroke.^[16]^ Therefore, it is reasonable to assume that there may be a synergistic interaction between VOCs and diabetes in precipitating stroke. Therefore, vigilant cardiovascular monitoring and appropriate health surveillance protocols are essential for diabetic patients.

Notably, inconsistent findings were observed between the WQS and BKMR models, which warrant careful interpretation. Although both methods are commonly used for mixture analysis, they rely on different statistical assumptions. WQS assumes a linear and unidirectional effect of mixture components and primarily captures additive relationships, whereas BKMR is a flexible, nonparametric approach that can model nonlinear, nonadditive, and interaction effects among exposures.^[17]^

These differences may explain why WQS did not identify a significant association in the overall population, while BKMR detected a positive association in the diabetic subgroup. When exposure–response relationships are complex, nonlinear, or vary across subpopulations, WQS may have limited ability to detect such patterns, whereas BKMR may be more sensitive to these effects.^[18]^

The association observed in the diabetic subgroup may also reflect increased susceptibility. Individuals with diabetes often exhibit oxidative stress and endothelial dysfunction, which could enhance vulnerability to environmental exposures such as VOCs. Similar patterns have been reported in mixture analyses, where stronger effects are observed in susceptible populations.^[19]^

Overall, these findings should be considered complementary rather than contradictory, highlighting the complexity of mixture effects. Interpretation should therefore account for the differing assumptions and strengths of each analytical approach.

A cohort study has identified 1,3-butadiene and acrolein as key characteristic metabolites in the primary sources of exposure of US residents.^[20]^ Personal exposure to VOCs has increased by 27%, with a significant positive association between mixtures of pollutants and all-cause mortality. This underscores the contribution of air pollution in the United States to the incidence of stroke and the escalating burden of disease. Urgent action is therefore needed to regulate VOCs, especially in relation to various daily necessities in both urban and rural areas.

The increased incidence of stroke in the population has been attributed to the influence of VOCs through multiple pathways and given the prevailing air pollution conditions in the United States, it is critical to strengthen regulatory measures in relevant industrial sectors and to reduce the risk of stroke by reducing atmospheric VOC concentrations.

The present study has several strengths. First, 2 mixture models, BKMR and WQS, were used to assess the association between VOC metabolite mixtures and stroke. This is a novel approach that has provided new insights into the effects of VOC metabolite mixtures on cardiovascular health. Mixture analysis is important because it can assess the joint effect of multiple VOCs, which may reflect more realistic exposure scenarios and thus provide a better understanding of the association between VOC metabolite mixtures and stroke. Second, no joint effect of VOCs was observed in either mixture model.

There are several limitations to this study. First, stroke was assessed by self-report questionnaires, which may have introduced bias. Importantly, such self-reported outcomes are subject to misclassification, particularly recall bias and diagnostic uncertainty. Prior epidemiological studies suggest that self-reported stroke tends to have moderate sensitivity but relatively high specificity; therefore, any misclassification is likely to be non-differential with respect to VOC exposure, which may bias the observed associations toward the null and potentially underestimate true effects. Second, the lack of a detailed classification of stroke subtypes in this study limited our understanding of the association between VOC metabolite mixtures and specific stroke subtypes. Therefore, caution should be exercised when interpreting these results and considering how different classification methods may affect the results. Third, due to the cross-sectional design of NHANES, exposure and outcome were measured simultaneously, precluding the establishment of temporal relationships. As a result, causal inference cannot be made, and reverse causation cannot be ruled out – for example, individuals with a history of stroke may have altered behaviors or environmental exposures that influence VOC metabolite levels. This limitation is well-recognized in cross-sectional environmental epidemiology studies. Fourth, although we adjusted for multiple known confounders, residual confounding may still exist. Certain factors, such as detailed occupational exposures, indoor air pollution sources, comorbidities severity, medication use, and healthcare access, were not fully captured or may have been measured with error. In addition, co-exposure to other environmental pollutants (e.g., particulate matter or heavy metals) may correlate with VOC exposure and influence stroke risk, potentially leading to biased estimates. Finally, the potential impact of multicollinearity among the measured chemicals is a critical consideration. As demonstrated by our correlation analysis (Fig. 1), several VOC metabolites exhibited moderate to strong positive correlations. To mitigate the risk of variance inflation and unstable estimates inherent in traditional multi-pollutant logistic regression, we specifically employed WQS and BKMR models. WQS effectively handles highly correlated components by estimating a unidirectional mixture effect, while BKMR utilizes a nonparametric kernel function to evaluate joint effects and address collinearity without the strict assumptions of generalized linear models. Despite the robust nature of these models, complex exposure patterns and residual multicollinearity may still partially affect the interpretability of individual effect estimates. Future studies should aim to overcome these limitations. Stroke subtypes should be further classified to better understand the effects of different VOC metabolite mixtures on different stroke types. Objective assessment of stroke may minimize the influence of self-reported data. Longitudinal studies are needed to verify causality and to investigate the long-term effect of VOC exposure on stroke risk. Improving our regulatory system to control VOC emissions could significantly reduce the incidence of stroke and improve public health.

## 5. Conclusions

In this cross-sectional study based on NHANES data, several urinary VOC metabolites, including CEMA, 3HPMA, and DHBMA, showed associations with stroke; however, these findings should be interpreted with caution given the observational nature of the study design. Importantly, the results were not fully consistent across analytical models, as mixture-based approaches (WQS and BKMR) did not demonstrate robust overall associations in the general population. Therefore, the observed relationships may reflect complex exposure patterns rather than independent or causal effects of specific VOCs. Overall, our findings provide preliminary epidemiological evidence suggesting a potential link between VOC exposure and stroke risk, particularly in certain subpopulations such as individuals with diabetes. Nevertheless, due to the limitations inherent in cross-sectional analyses and potential residual confounding, no causal inferences can be drawn. Further well-designed longitudinal studies are required to validate these findings and clarify the underlying mechanisms.

## Acknowledgments

The authors would like to thank all participants who participated in the study.

## Author contributions

**Conceptualization:** Hanguang Liu.

**Data curation:** Hanguang Liu.

**Formal analysis:** Hanguang Liu.

**Funding acquisition:** Hanguang Liu.

**Methodology:** Xiaoying Zhu.

**Project administration:** Xiaoying Zhu.

**Resources:** Xiaoying Zhu.

**Software:** Xiaoying Zhu.

**Writing – original draft:** Hanguang Liu.

**Writing – review & editing:** Xiaoying Zhu.


