# Supplementary figures and images for: Urinary volatile organic compounds and stroke risk: A cross-sectional analysis of NHANES data
Source: Medicine (Baltimore). 2026 May 15;105(20):e48786. doi: 10.1097/MD.0000000000048786 (PMC13183055; doi:10.1097/MD.0000000000048786)

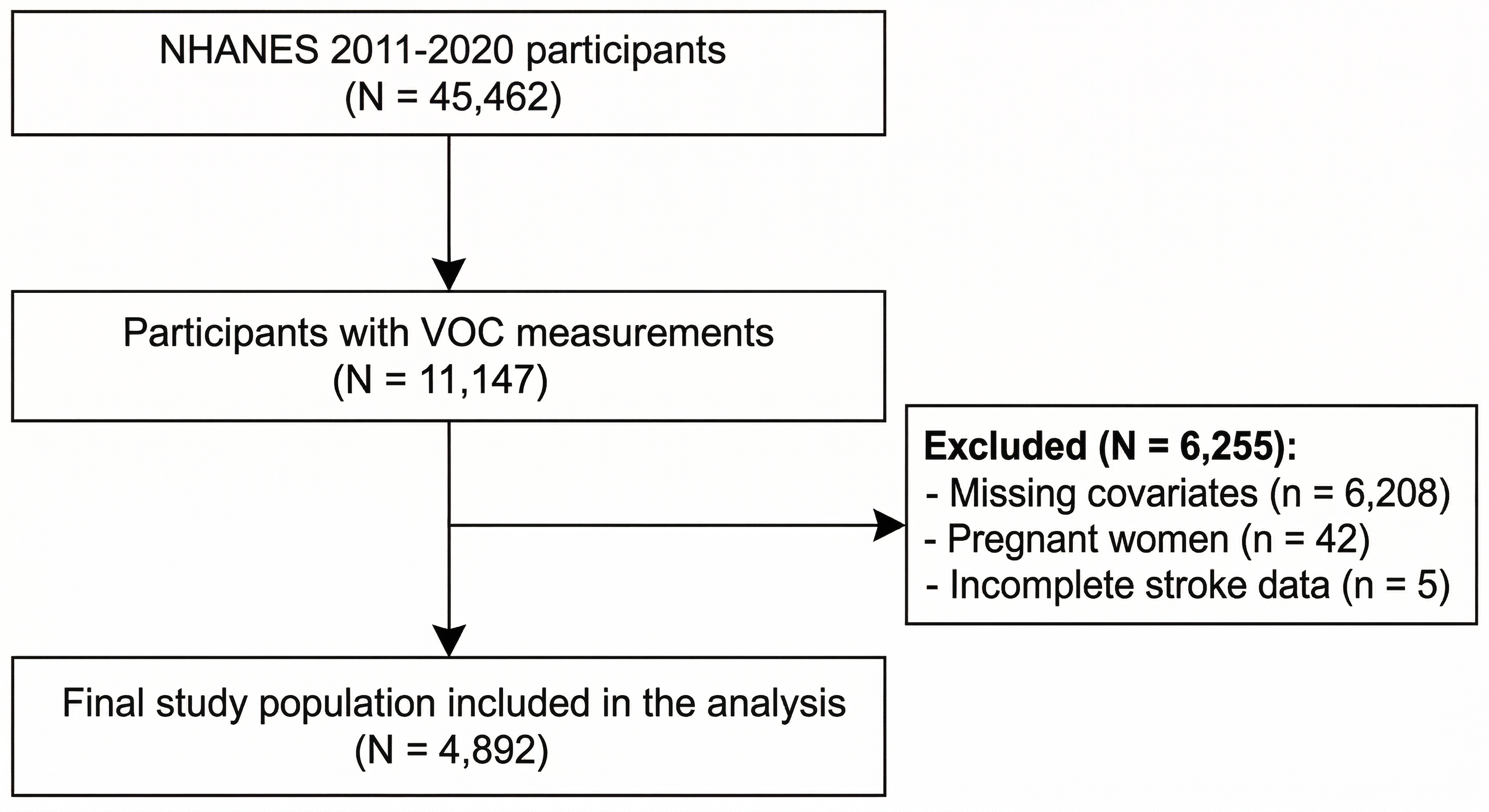


Supplementary Figure 1. Flow diagram of the participant selection process

Supplement: Supplementary file 1 [file medi-105-e48786-s001.docx]
